# Scabies complicated by necrotizing lymphocytic vasculitis in an infant

**DOI:** 10.11604/pamj.2020.36.166.24431

**Published:** 2020-07-09

**Authors:** Houda Nassih, Fatimzehra El Hanafi, Safa Elalaoui, Rabiy El Qadiry, Aicha Bourahouat, Imane Ait Sab

**Affiliations:** 1 Pediatric Ward, Department of Pediatrics, Marrakesh Medical and Pharmacy Faculty, Child and Mother Hospital, Mohammed VI University Hospital Center, Caddy Ayad University, Marrakesh, Morocco

**Keywords:** Scabies, infant, necrotizing lymphocytic vasculitis, acral necrosis

## Abstract

Scabies is very common among children. It is often a harmless infectious disease, responding well to antiparasitic medication. Nevertheless, severe forms can occur in immunocompromised populations like newborns and infants. We report a unique case of scabies in a three-months-old infant, complicated by generalized cutaneous lymphocytic vasculitis and unilateral acral necroses.

## Introduction

Lymphocytic vasculitis is a morphological term that includes clinically heterogenous diseases like connective tissue disease, infection, lichenoid diseases, drug reaction, Bechet's disease, superficial thrombophlebitis and leukemic vasculitis. Necrotizing forms are possible. Among infectious causes, our case is the first pediatric case where scabies is incriminated. Moreover, acral necroses occurred and imputation was necessary.

## Patient and observation

**Figure 1:**
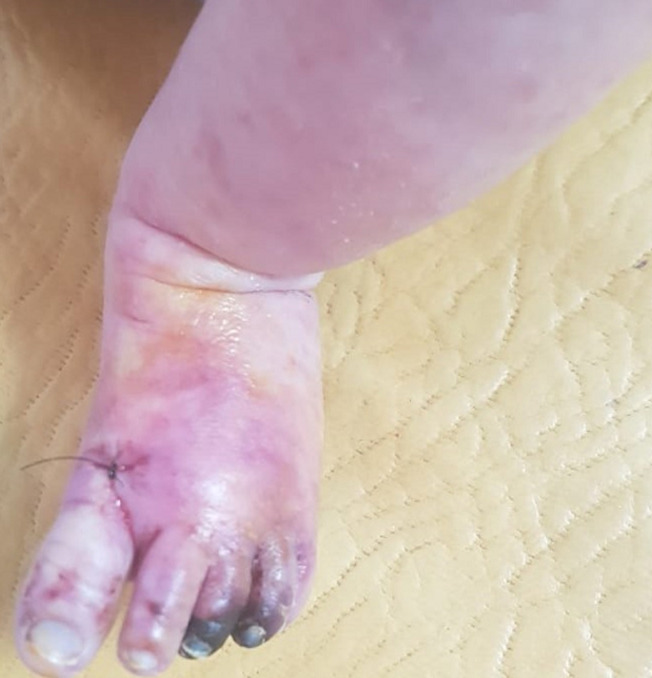
a three months-old infant with acral necroses of the third and fourth left toes

**Figure 2:**
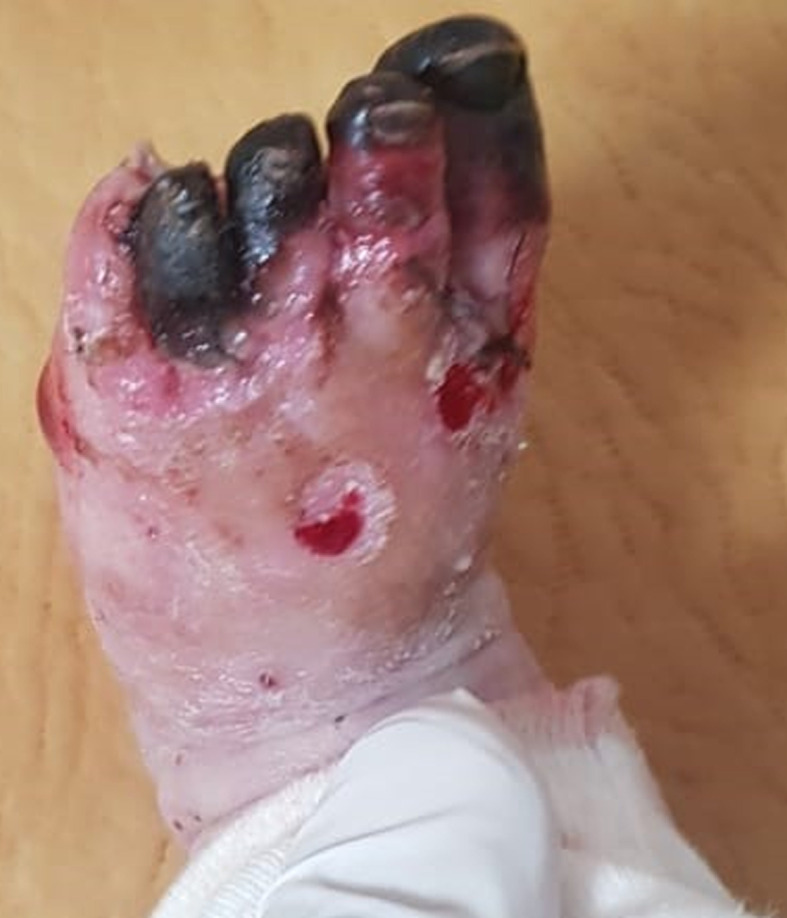
acral necroses of all toes of the left foo

A well-appearing three-month-old boy, with no past medical history, was admitted to the emergency department for an acute onset of ischemia of the left foot evolving for twelve hours. The mother reported a generalized rash evolving for one month. The boy was under no medication. Both parents suffered from generalized scratching. At the examination, the infant was afebrile and had severe itchiness and a pimple-like rash typical of scabies. The presence of pustules suggested additional bacterial infection. Swab sampling was performed and methicillin-sensitive *Staphylococcus aureus*was isolated on culture. The child was put on oral antibiotics (amoxicillin + clavulanic-acid: 80 mg/kg/day, three times a day, for 10 days) and benzyl benzoate emulsion. Parents´ treatment, as well as decontamination of clothes and blankets were started. The boy had also necrosis of the third and fort toes of the left foot (Figure 1). Peripheral pulses of the four limbs were well perceived. Doppler of the left limb did not visualize any thrombus. A typical rush consisting of vasculitic elements was also found on skin examination. Biopsy and pathology examination found a lymphocytic vasculitis of the dermis and hypodermis. There was no IgG, IgM, IgA or C3 perivascular deposits on immunofluorescence. The next day after admission, the infant developed acrocyanosis of the left foot and necroses of all five toes (Figure 2).

We started aspirin at 5mg/kg/day. Abdominal and lower limbs CT-angiography was normal. There was a mild inflammatory syndrome (CRP = 50.86mg/l, ESR = 34mm, ferritin=109ng/ml, ANC = 13230/mm^3^, platelets = 760000/mm^3^) that was most likely due to infection. Laboratory workup to look for thrombophilia and deep vein thrombosis was normal (PT = 100%, PTT = 32sec, Fibrinogen = 1.92g/l, D-Dimer = 0.66ng/ml, C protein = 72%, S protein = 70%, antithrombin III = 81%). Also, cryoglobulinemia blood essay was negative. Capillaroscopy found no abnormalities of the microcirculation in the nail folds of the right foot. We looked also for an underlying auto-immune condition. Plasma protein electrophoresis was normal and the coombs test was negative. Antinuclear antibodies were weakly positive and speckled. Anti-ds DNA antibodies, anti-Sm antibodies, anti-Ro/La antibodies, anti-RNP antibodies, anti-P-ANCA/C-ANCA antibodies, anti-cardiolipin and anti-beta-2 glycoprotein 1 antibodies were negative. No lupus anticoagulant was found. Evolution was marked by regression of acrocyanosis by the third day. Acral ischemia progression stabilized. Amputation of the five toes was performed by the seventh day. Meanwhile, scabies lesions have healed by the second week and CRP decreased to 17.1mg/l. After what, the boy was discharged home. He is now five-month-old and still healthy.

## Discussion

Lymphocytic vasculitis (LV) is usually arbitrarily defined by different authors as to have lymphocytes attacking a small vessel, endothelial swelling with or without fibrin deposition [1]. Infections have associated with or been attributed to the presence of vasculitis in a variable proportion of patients [1]. Many different viruses have been implicated including hepatitis B and C, as well as several bacterial infections including streptococcal infections, other causes of subacute bacterial endocarditis, meningococcal infection and tuberculosis (associated with some cases of nodular vasculitis) [2]. The pathogenetic mechanisms involved are mainly immunological, immune complex-mediated tissue injury being the most commonly incriminated factor. Differential diagnoses are lupus erythematosus, lymphoma and pityriasis lichenoides associated LV [3]. Management of bacterial LV consists mainly of antibiotics [4]. Also, bacterial seeding of vessels may lead to necrosis through direct bacterial action. In exceptional cases, acral necrosis can occur, an amputation might be necessary [5]. No pediatric case associating scabies to LV has been described to date. The main cause of LV in our infant is not yet clear. We tend to believe it is staphylococcal infection rather than scabies.

## Conclusion

Lymphocytic vasculitis is rare and not yet well understood, with a full list of possible causes that have yet to be assembled. The prognosis of lymphocytic vasculitis depends on the extent and severity of the disease. In most cases, it affects only the skin and can be a mild benign condition that eventually heals.
